# Ahead of the Curve: Responses From Patients in Treatment for Obsessive-Compulsive Disorder to Coronavirus Disease 2019

**DOI:** 10.3389/fpsyg.2020.572153

**Published:** 2020-10-27

**Authors:** Jennie M. Kuckertz, Nathaniel Van Kirk, David Alperovitz, Jacob A. Nota, Martha J. Falkenstein, Meghan Schreck, Jason W. Krompinger

**Affiliations:** ^1^Obsessive Compulsive Disorder Institute, McLean Hospital, Belmont, MA, United States; ^2^Department of Psychiatry, Harvard Medical School, Boston, MA, United States

**Keywords:** obsessive compulsive disorder, anxiety, COVID-19, coronavirus, acceptance and commitment therapy, exposure and response prevention, resilience

## Abstract

Alongside concern about the physical health impacts of the coronavirus disease 2019 (COVID-19) crisis, public health officials have also raised concerns about the potential for massive mental health impact. This has led many to wonder, how are individuals with obsessive-compulsive disorder (OCD), and especially those with contamination fears, doing in the era of COVID-19? We present data from eight patients in our residential treatment program for OCD who were admitted prior to any COVID-19 restrictions and continued in treatment at the facility during the pandemic. Much like the general population, our patients varied in the ways they were impacted by COVID-19, yet the majority experienced improvements in OCD symptoms despite the context. This is not to downplay the many ways in which our patients were personally affected by COVID-19. Rather our patients’ relatively resilient responses mirror our program’s treatment model, which emphasizes exposure and response prevention (ERP) within the complementary framework of acceptance and commitment therapy (ACT). The intention of this article is to challenge the notion that by definition this population will fare worse than the general public or that ERP cannot proceed effectively during this time. In contrast, we underscore that effective OCD treatment can and should continue in the era of COVID-19.

## Introduction

Coronavirus disease 2019 (COVID-19) was identified on December 31, 2019 and has become a global pandemic (World Health Organization, 2020) that has resulted in over 4 million positive cases and over 146,000 deaths in the United States alone (Centers for Disease Control and Prevention, 2020). Alongside physical health concerns, public health officials have raised concerns about the potential for massive mental health impact based on increased stress associated with fear of contracting/transmitting the virus and substantial changes to routine, financial ramifications, and social isolation (Holmes et al., 2020; Pfefferbaum and North, 2020; Yao et al., 2020). Initial data validate concerns regarding these negative mental health impacts among the general population (Ipsos MORI, 2020; Qiu et al., 2020), medical workers (Lu et al., 2020), college students (Cao et al., 2020), and individuals living in regions heavily impacted by COVID-19 (Liu et al., 2020).

This has led many to wonder, how are individuals with obsessive-compulsive disorder (OCD), and especially those with contamination fears, doing in the era of COVID-19? Myriad articles written by popular press and professional organizations have speculated about this topic and offered suggestions (Anxiety and Depression Association of America, 2020; Fontenelle and Miguel, 2020; International OCD Foundation, 2020; Rosman, 2020). The authors of the current article are clinicians and researchers at a residential treatment program for severe OCD. Frequently, we have heard comments from colleagues in the broader psychology and health fields such as “must be an interesting time to work with OCD,” “your poor patients must be really struggling,” or “how do you even do treatment right now when everyone has OCD?” The implication is that people with OCD are especially struggling to cope with the current COVID-19 realities, even for individuals currently in treatment.

To some degree, these assumptions are intuitive given that difficulty tolerating uncertainty (which is highly salient in this unprecedented global pandemic) is a hallmark feature of OCD (Obsessive Compulsive Cognitions Working Group, 2005) and 40–50% of individuals with OCD report concerns about germs or contamination (Pinto et al., 2008; Matsunaga et al., 2010). Moreover, existing literature suggests that obsessive-compulsive contamination and/or health anxiety symptoms are associated with greater anxiety about prior public health concerns among university-affiliated samples (Wheaton et al., 2012; Blakey et al., 2015; Blakey and Abramowitz, 2017).

However, the impact of the current pandemic on individuals with a diagnosis of OCD remains unclear. COVID-19 is unprecedented in modern history in its scope and impact on daily routine and behaviors (Callaway et al., 2020; Pew Research Center, 2020). Given recent public health directives to wash hands, sanitize items, and monitor symptoms frequently, it is reasonable to hypothesize that obsessive-compulsive symptoms might increase for individuals in the community who do not ordinarily engage in these behaviors. What is unclear is to what extent the current global pandemic results in a clinically meaningful exacerbation of OCD among individuals who already struggle with these symptoms in the absence of a pandemic. More importantly, in our minds, is the question of whether it is possible for individuals with OCD to cope adaptively (e.g., by engaging in treatment) during this time.

The assumption that all patients with OCD are uniquely struggling is discordant with our experiences working within a treatment context. We are not alone in our anecdotal impressions that many patients are doing well (Rosmarin, 2020) and that individuals who have engaged in treatment for OCD may be uniquely well-positioned to weather the COVID-19 storm with resilience (Morse, 2020). At the broader societal level, it has been noted that COVID-19 has the potential for positive impacts on mental health and wellbeing, including increased time for exercise, healthy eating, family, and friends (Delgado, 2020).

Given these mixed hypotheses about the impact of COVID-19 on the population we serve, we present both quantitative and qualitative data from eight patients in our residential treatment program. Given the unforeseen nature of this crisis, we did not (nor could we have) systematically design the optimal research methodology to study this question. We acknowledge that ours is not necessarily a representative nor random sample, as we were required to sharply reduce our census, and discharge decisions were based on geography, patients’ desires to remain in treatment, and perceived ability to benefit. Nonetheless, rather than leave to speculation, our goal was to bring the empirical data that we do have to bear on the question of how patients in residential treatment for OCD have responded to the ongoing pandemic.

As such, we present data from patients who were (1) admitted prior to COVID-19 restrictions (January 6, 2020–February 24, 2020) and had the experience of our program per usual, and (2) continued in the program throughout a number of COVID-19 impacts (discharged April 7, 2020–May 22, 2020), including a no-visitor policy, being required to stay on unit, mask requirements for patients and staff, changes to meals and their delivery, significant reduction in census to maintain social distancing, and news of confirmed positive COVID-19 staff cases. Typical programming included 2–4 h daily of exposure and response prevention (ERP), four groups daily based on cognitive-behavioral and acceptance and commitment therapy (ACT), and meetings with a behavior therapist (2–3x/week), a family therapist (1x/week), and a psychiatrist (1x/week). Average length of stay was 83.9 days (*SD* = 17.2, range = 58–106). Patients completed weekly measures of OCD severity (Yale-Brown Obsessive Compulsive Scale, YBOCS; Goodman et al., 1989), quality of life (Quality of Life Enjoyment and Satisfaction Questionnaire, QLES; Endicott et al., 1993), and worry (Penn State Worry Questionnaire-Abbreviated, PSWQ-A; Crittendon and Hopko, 2006). Patients also completed the Dimensional Obsessive–Compulsive Scale at admission to characterize symptom presentation(s) (Abramowitz et al., 2010). Data were collected *via* a larger project that received institutional review board approval and for which patients provided informed consent.

## How Have Patients in Residential Treatment for OCD Fared in the Era of COVID-19?

**Figure 1:**
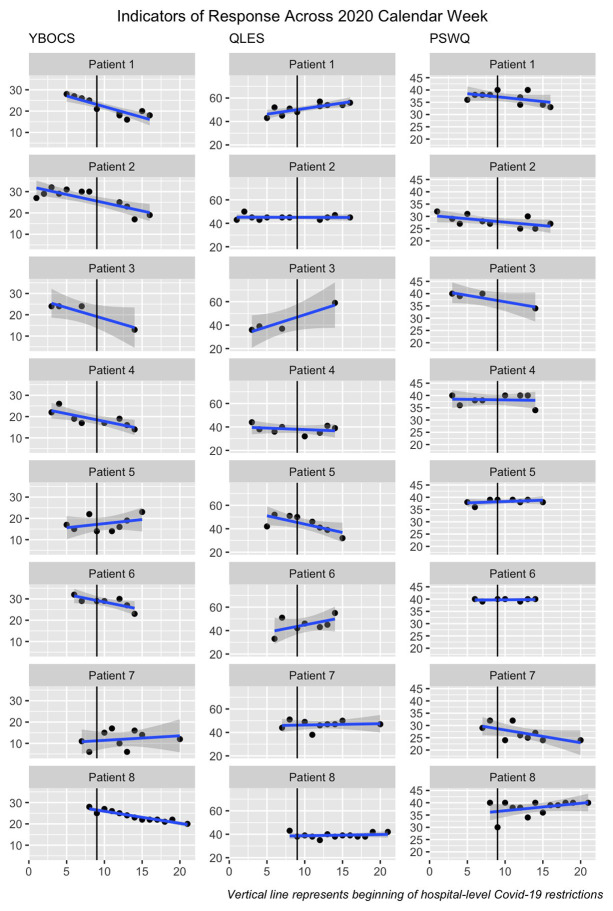
Indicators of response across 2020 calendar week. YBOCS, Yale-Brown Obsessive Compulsive Scale; QLES, Quality Of Life Enjoyment and Satisfaction Questionnaire; PSWQ, Penn State Worry Questionnaire-Abbreviated. Data were visualized with *ggplot2* in R (Wickham et al., 2016).

Mean YBOCS at admission was 23.62 (*SD* = 6.82, *N* = 8), corresponding to moderate to severe OCD. Figure 1 displays patient-reported OCD severity, quality of life, and worry across treatment and suggests that most individuals experienced OCD symptom improvement despite the context of COVID-19. Changes in quality of life and worry were variable across and within patients. Follow-up data were available for five patients (*M* = 36.4 days after discharge, *SD* = 8.7, range = 35–49) and generally suggested stability of treatment effects (Table 1).

**Table 1:** Indicators of treatment response and baseline symptom presentation.

	YBOCS			QLES			PSWQ			DOCS (Baseline only)				
Patient	BAS	END	FU	BAS	END	FU	BAS	END	FU	Total	Cat 1	Cat 2	Cat 3	Cat 4
Patient 1	28	18	18	43	56	56	36	33	33	54	11	7	17	19^*^
Patient 2	27	19	18	43	45	46	32	27	27	14	13^*^	0	1	0
Patient 3	24	13	--	36	59	--	40	34	--	25	0	10	15^*^	0
Patient 4	22	14	15	44	39	42	40	34	36	30	7	0	13^*^	10
Patient 5	17	23	--	42	32	--	38	38	--	24	11^*^	2	9	2
Patient 6	32	23	22	33	55	48	40	40	40	60	17	20^*^	15	8
Patient 7	11	12	--	44	47	--	29	24	--	14	11^*^	0	0	3
Patient 8	28	20	8	43	42	40	40	40	34	58	15	15	16^*^	12
*M*	23.6	17.8	16.2	41.0	46.9	46.4	36.9	33.8	34.0	34.9	10.6	6.8	10.8	6.8
*SD*	6.8	4.3	5.2	4.1	9.3	6.2	4.3	5.8	4.7	19.4	5.2	7.7	6.8	6.7

* indicates primary symptom domain.

YBOCS, Yale-Brown Obsessive Compulsive Scale; QLES, Quality Of Life Enjoyment And Satisfaction Questionnaire; PSWQ, Penn State Worry Questionnaire-Abbreviated; DOCS, Dimensional Obsessive-Compulsive Scale; comprises four categories. Category 1: Concerns about germs and contamination [shaded due to potential relevance to coronavirus disease 2019 (COVID-19)]. Category 2: Concerns about being responsible for harm, injury, or bad luck. Category 3: Unacceptable thoughts. Category 4: Concerns about symmetry, completeness, “just right.” BAS, baseline; END, endpoint; FU, follow up. DOCS are presented for baseline only.

To further support our descriptive, visual, and qualitative data, we examined slopes of each indicator using mixed models to provide quantitative, group-level measures of effect. Mixed models are advantageous for examining longitudinal data in small samples relative to other analytic methods because they maximize power (Muth et al., 2016) and were examined using *nlme* in R (Pinheiro et al., 2019). Similar to individual-level visualization, these group-level analyses indicated that patients’ OCD symptoms declined [*B* = −0.50, SE = 0.15, *t*(64) = −3.42, *p* = 0.001]. On average, there were no changes in quality of life [*B* = 0.30, SE = 0.30, *t*(64) = 1.02, *p* = 0.313] or worry [*B* = −0.14, SE = 0.09, *t*(63) = −1.47, *p* = 0.147].

To provide a rough context for how patients in our program respond in the absence of a pandemic and to account for seasonal effects on mental health (Tan et al., 2017), we examined the same indicators among patients (*N* = 10) who admitted and discharged in the comparable 2019 calendar weeks. Mean YBOCS at admission was 27.50 (*SD* = 6.59). Mixed models indicated that patients’ OCD symptoms declined [*B* = −0.70, SE = 0.18, *t*(95) = −3.93, *p* < 0.001]. On average, there were no changes in patients’ quality of life [*B* = 0.24, SE = 0.15, *t*(90) = 1.56, *p* = 0.122] but worry did decrease [*B* = −0.38, SE = 0.07, *t*(87) = −5.06, *p* < 0.001].

## Individual Case Vignettes

To contextualize individual-level data (Table 1; Figure 1), we briefly describe each patient’s treatment trajectory and response to COVID-19.

### Patient 1

This patient presented with symmetry and exactness concerns, perfectionism, harm obsessions, and need to understand. For years, they had avoided phone calls or videoconferencing because of fears of not being able to “control the content.” The increasing emphasis on using phone/videoconferencing for treatment amid the pandemic was both highly triggering for this patient and provided motivation to address this issue. This patient reported increased anxiety when staff and patients were required to wear masks, as it triggered their end-of-the-world obsessions, but they were able to implement appropriate coping skills and reported feeling happy about socializing with peers despite masks. By discharge, the patient was able to consistently use audio/video communication for treatment and socially, reporting feeling happy that they were able to connect with their support system in these ways.

### Patient 2

This patient presented with contamination symptoms around exposure to everyday chemicals that may alter their existence. The patient expressed little concern about contracting COVID-19 although found that the requirement to increase use of hand sanitizer and soap provided a push toward exposure, as these substances were triggers. The patient reflected that they noticed their parent, who does not have OCD, engaging in behaviors that appeared reassurance seeking and ruminative, and found it interesting that they were able to provide feedback to their parent about the function of these behaviors. This patient continued to make treatment progress before and during the onset of COVID-19 changes.

### Patient 3

This patient presented with harm obsessions, perfectionism, scrupulosity, social anxiety, and eating disordered symptoms around food and exercise. The onset of COVID-19 elevated both normative and OCD worries, but also provided opportunities for fuller engagement with treatment goals. This patient’s employer was affected by the pandemic, and they reported worrying about how coworkers would pay bills. Upon learning that a unit staff member tested positive, the patient experienced anxiety about not knowing who the staff member was (due to privacy policies) and about potential exposure to that staff member. The patient was anxious about receiving pre-packaged meals and not being able to go to the gym, but was able to be more flexible around these behaviors. Their clinicians noted that the patient remained fully engaged in treatment despite these increased anxieties and spent more time thinking about how to engage in activities with meaning and enjoyment.

### Patient 4

This patient presented with harm obsessions and contamination fears resulting in vomiting. Additionally, not-just-right experiences, superstitious obsessions, and agoraphobia symptoms were endorsed. As their ultimate feared consequence within the contamination realm centered on illnesses causing vomiting, COVID-19 was not significantly triggering (beyond the universal anxiety associated with navigating the pandemic). As the pandemic progressed and unit restrictions intensified, the patient was unable to continue public transportation exposures. Even as these restrictions were implemented, they continued to make significant progress and refocused their exposures to target symptoms around vomiting, harm, and food-related obsessions.

### Patient 5

This patient presented with concerns around perfectionism, intrusive thoughts, and contamination. The patient reported COVID-19 related stressors and increases in anxiety throughout the pandemic, including feeling hyper-aware of physical symptoms, worry that their partner would be less available due to pandemic-related increased work hours, learning that coworkers had been laid off, and having family members with the virus become seriously ill. The patient reported that these concerns caused them to feel distracted, cry, and experience difficulty sleeping. Nonetheless, throughout this time, exposure coaches rated the patient as highly engaged in perfectionism-related and interoceptive exposure exercises.

### Patient 6

This patient presented with fears of rejection, intrusive thoughts, emetophobia, and panic symptoms. When the program reduced patient census due to COVID-19 restrictions, the patient expressed sadness and increased panic due to their closest peers discharging and concern that they would not have people with whom to connect and practice being vulnerable. They described feeling overwhelmed by the impact of COVID-19 on their community, including family members becoming ill. Nonetheless the patient identified positive ways to engage with family and valued activities to maintain structure following discharge and generally remained focused on treatment.

### Patient 7

This patient presented with primary skin picking disorder in the context of family stressors along with a variety of “not just right” experiences. The patient exhibited an increase in skills and a decrease in skin picking over the first 3 weeks in treatment. The patient and treatment team were beginning to plan for exposures in the patient’s home, but these were paused due to COVID-19 restrictions. Given this limitation and throughout various COVID-19 related changes, this patient expressed concern that they may not receive optimal treatment and considered discharging and returning post-COVID-19 yet ultimately decided to stay. The patient discharged 1 week prematurely due to exhibiting potential symptoms of COVID-19. Overall, the patient made good progress despite setbacks during stressful events and notably completed home-based exposures *via* a newly-developed virtual treatment program following discharge.

### Patient 8

This patient presented with harm and contamination symptoms, including fear of bodily fluids and contracting disease. The patient progressed through treatment relatively fluidly despite COVID-19 related changes to their plan. For example, the patient discontinued exposures of brushing against people in crowded areas and shifted instead to similar imaginal exposures. Rather than resisting hand washing following exposure to household surfaces, the patient practiced washing their hands for 20 s and then moving forward to other activities. The patient’s therapist noted that the patient “does not seem overtly concerned about the coronavirus” even after another member of the patient’s treatment team tested positive. The patient mentioned feeling as though the social connection and activities provided through the program structure were helpful in managing symptoms.

## Conclusion

Much like the general population, our patients with OCD varied in the ways they were impacted and responded to emerging COVID-19 related events. For some individuals, COVID-19 actually provided opportunities or motivation to more fully engage in exposure (Patients 1, 2) or other treatment goals (Patient 3). At the same time, some patients did encounter COVID-19 related exacerbation of symptoms (Patient 5) or required modifications to their treatment plan due to increased restrictions (Patients 4, 7, and 8). Most commonly, however, patients experienced COVID-19 related stressors due to general societal, familial, and economic consequences of a global pandemic (Patients 3, 5, 6, and 7). For these reasons, it is perhaps unsurprising that patients did not on average experience a significant reduction in worry during COVID-19 (in contrast to treatment effects for 2019 data).

Overall, our data do not support the notion that our patient population uniquely and universally struggled in the face of COVID-19. This is not surprising to us as clinicians. Common refrains we heard echoed from patients at the peak of COVID-19 disruptions were “this is what we’ve been training for!” and “we have other things that we’re worried about.” Typically said in a lighthearted way, these statements are illustrative of how our patients have responded: with appropriate in-group humor, fostering a “we’re all in this together” attitude, with resilience and acceptance of the present realities. This is not to downplay the ways our patients were personally affected by COVID-19 nor their corresponding experiences of anxiety, fear, and sadness, or the fact that COVID-19 can exacerbate OCD symptoms when aligning with one’s obsessional content.

Our patients’ responses may mirror our program’s treatment model, which emphasizes ERP within the complementary framework of ACT (Twohig et al., 2018). The emphasis in ACT is on having inner experiences without trying to control or push them away (Twohig, 2009). Instead, we can choose to engage in value-driven behaviors despite the context of difficult thoughts and feelings (Twohig, 2009). Through ERP, our patients learn to intentionally approach situations that trigger anxiety and uncertainty with curiosity and openness, with an overarching clinical goal to foster resiliency and flexibility. We have seen our patients embrace this idea even (especially) in the era of COVID-19 in how they utilize their time in treatment and plan for return home amid ongoing restrictions.

There has been much discussion of potential silver linings of COVID-19 at the societal level (Delgado, 2020), and this was true for some of our patients, such as increased opportunities for exposure to avoided situations. Data on the extent to which silver linings have come to fruition in the general public has been mixed (Gallup, 2020). While our patients reported mixed impacts, it is notable that we did not observe massive or consistent declines in quality of life. Most patients did see continued improvements in OCD symptoms, underscoring that effective OCD treatment can and should continue despite COVID-19 (Krompinger et al., 2020).

We appreciate that questions about how our patients are doing during COVID-19 typically stem from a place of caring and concern. So too, however, must we be aware of stigma and implicit assumptions that our patients with OCD may fall apart or cease to function adaptively during this time. It is important to acknowledge the resilience our patients possess, and recognize that the treatment principles patients must master to overcome their symptoms uniquely position them to cope with situations of unprecedented uncertainty. By acknowledging these facts, we challenge the potential effects of stigma, such as relegating those with OCD to the “sick” or “fragile” role.

It is important to note that our patients were engaged in intensive treatment and thus received significant support and in-person socialization. For some patients, their initial targeted avoidance behaviors (i.e., hand washing) were now prescribed by the CDC and as such, adaptations made to their plans on the fly were thoughtful and in keeping with the underlying principles of ERP. Thus, our setting provides an optimal context and we do not mean to imply that our findings generalize to all individuals with OCD. Rather, we challenge the notion that by definition this population will fare worse than the general public or that ERP treatment cannot proceed effectively because “everyone has OCD.”

This pandemic is not over, and continued research on patient longer-term responses is being conducted by our group and others. Meanwhile, we recommend that clinicians continue to encourage individuals with OCD to seek treatment. Given the benefit of social support from other individuals with OCD, we also encourage people with these symptoms to access any number of clinician-led or peer-led support groups available online. The International OCD Foundation^1^ is a good place to start.

## Data Availability Statement

The raw data supporting the conclusions of this article will be made available by the authors, without undue reservation.

## Ethics Statement

The studies involving human participants were reviewed and approved by Partners Healthcare System Institutional Review Board. The patients/participants provided their written informed consent to participate in this study. Written informed consent was obtained from the individual(s) for the publication of any potentially identifiable images or data included in this article.

## Author Contributions

JMK wrote the initial draft of the manuscript, analyzed the quantitative data, and incorporated co-author feedback. All authors provided treatment for the patients described in the manuscript and contributed to the conceptualization of the manuscript. NK and MS contributed to writing the case vignettes. NK, DA, MF, and JN provided written feedback on manuscript drafts. NK, DA, and MF contributed to the introduction and conclusion. All authors contributed to the article and approved the submitted version.

### Conflict of Interest

The authors declare that the research was conducted in the absence of any commercial or financial relationships that could be construed as a potential conflict of interest.

## Footnotes

^1^https://iocdf.org/
